# JKA97, a Novel Benzylidene Analog of Harmine, Exerts Anti-Cancer Effects by Inducing G1 Arrest, Apoptosis, and p53-Independent Up-Regulation of p21

**DOI:** 10.1371/journal.pone.0034303

**Published:** 2012-04-27

**Authors:** Xinyi Yang, Wei Wang, Jiang-Jiang Qin, Ming-Hai Wang, Horrick Sharma, John K. Buolamwini, Hui Wang, Ruiwen Zhang

**Affiliations:** 1 Department of Pharmaceutical Sciences, School of Pharmacy, Texas Tech University Health Sciences Center, Amarillo, Texas, United States of America; 2 Cancer Biology Center, School of Pharmacy, Texas Tech University Health Sciences Center, Amarillo, Texas, United States of America; 3 Department of Biomedical Sciences, School of Pharmacy, Texas Tech University Health Sciences Center, Amarillo, Texas, United States of America; 4 Department of Pharmaceutical Sciences, College of Pharmacy, University of Tennessee Health Science Center, Memphis, Tennessee, United States of America; 5 Institute for Nutritional Sciences, Shanghai Institutes for Biological Sciences, Chinese Academy of Sciences, Shanghai, China; Wayne State University, United States of America

## Abstract

JKA97, a benzylidene analog of harmine, has been found to be a promising drug candidate for human cancer therapy, although the underlying molecular mechanisms have not been fully demonstrated. In this study, we evaluated the effects of JKA97 on human breast cancer cells *in vitro* and *in vivo*. JKA97 inhibited the growth and proliferation of MCF7 (p53 wild-type), MCF7 (p53 knockdown), and MDA-MB-468 (p53 mutant) cells in a dose-dependent manner. Treatment with JKA97 arrested breast cancer cells in G1 phase and induced apoptosis. JKA97 also significantly suppressed the growth of MCF7 and MDA-MB-468 xenograft tumors. It regulated the expression levels of G1 phase regulators, such as p21, p27, cyclinE, and cylinD1. JKA97 activated p21 transcription, independent of p53, but had little effect on p21 protein stability/degradation. In summary, our results suggest that JKA97 inhibits human breast cancer cell growth through activating p21, independent of p53, which provides a basis for developing this compound as a novel drug for human breast cancer therapy.

## Introduction

Breast cancer is one of the most commonly diagnosed cancers and the leading causes of cancer death in women worldwide. According to statistical data from GLOBOCAN, about 1.38 million new patients were diagnosed with breast cancer and about 458,400 died worldwide from the disease in 2008 [1]. In the past two decades, death rates from breast cancer have declined due to early detection, increased awareness and improved or novel therapies. However, certain forms of breast cancer are particularly aggressive and present a poor prognosis. Patients with advanced-stage or relapsed breast cancer often show modest response to clinically existing therapies and may even exhibit resistance to conventional chemotherapeutic agents [2]–[4]. The paucity and inefficacy of practicable therapeutics makes successful treatment of breast cancer a challenge and necessitates the discovery of alternative chemotherapeutic drugs with novel anti-cancer mechanisms.

Natural product-oriented synthetic derivatives have played an important role in anti-cancer drug discovery [5], [6]. Several widely-used chemotherapeutic agents such as paclitaxel and actinomycin C were originally developed from natural products. Harmine, a β-carboline alkaloid obtained from the seeds of the poisonous plant *Peganum harmala,* has been used medically for some time as an anti-hypertensive drug; acting by reversibly inhibiting monoamine oxidase A. Studies in the past decade revealed that harmine also possessed potent anti-proliferative and cytotoxic properties [7], [8].

**Figure 1:**
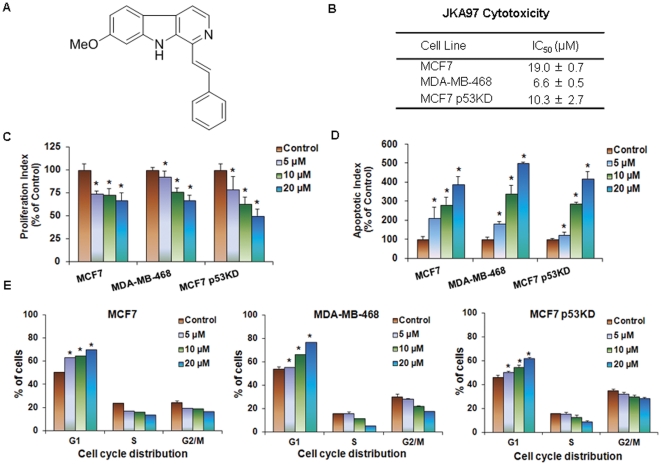
*In vitro* anticancer activities of JKA97 against breast cancer cells. (A) Chemical structure of JKA97. (B) Concentrations of JKA97 inducing 50% growth inhibition (IC_50_) in breast cancer cells, relative to the corresponding controls, based on the MTT assay. MCF7, MDA-MB-468, and MCF7 p53KD cells were exposed to various concentrations of JKA97 for 72 hrs. (C) Anti-proliferative effects of JKA97 on breast cancer cells. Cells were exposed to various concentrations of JKA97 for 48 hrs, followed by the BrdUrd incorporation assay. The proliferation index was calculated against untreated control cells (*P<0.05). (D) Induction of apoptosis in breast cancer cells by JKA97. Cells were exposed to various concentrations of JKA97 for 24 hrs, followed by measurement of apoptosis by Annexin V assay. The apoptotic index was calculated against untreated control cells (*P<0.05). (E) Effects of JKA97 on the cell cycle distribution of breast cancer cells. Cells were exposed to various concentrations of JKA97 for 24 hrs, followed by measurement DNA contents by flow cytometry. The cell cycle distribution was evaluated by comparing with that of control cells (*P<0.05). All the assays were performed in triplicate. Results were from at least three separate, repeated experiments.

JKA97, also referred as methoxy-1-styryl-9H-pyrido-[3,4-b]-indole (MW: 286.35; Fig. 1A), is a benzylidene analog of harmine [9], [10]. A recent report showed that JKA97 could induce apoptosis of colon and liver cancer cells *in vitro* and *in vivo* by a Bax-dependent and p53-independent mechanism [10]. However, the anti-cancer activities of JKA97 on other types of cancer and the precise molecular mechanisms of the compound are not well understood. In the present study, we explored the anti-tumor activities of JKA97 against breast cancer cells with various genetic backgrounds, and attempted to further elucidate the possible mechanisms of action, providing a basis for future development of this agent as human breast cancer therapy.

## Results

### JKA97 decreases breast cancer cell growth *in vitro*

JKA97 was evaluated for its effects on breast cancer cell viability *in vitro* by use of the MTT assay. Three human breast cancer cell lines representing three different genetic backgrounds (MCF7/p53 wild-type; MCF7/p53 knockdown, and MDA-MB-468/p53 mutant) were exposed to various concentrations of the test compound (0, 1, 2.5, 5, 10, 25, and 50 µM) for 72 hrs, and cell survival percentages were determined. As seen in Fig. 1B, the compound demonstrated IC_50_ values of less than 20 µM (6.6–19.0 µM). The MDA-MB-468 and MCF7 p53KD cells appeared to be more sensitive to the compound than MCF7 cells.

### JKA97 inhibits breast cancer cell proliferation *in vitro*

Similar to the effects on cell survival, JKA97 inhibited cell proliferation in a dose-dependent manner (Fig. 1C). Significant anti-proliferative effects were seen in all three cell types with various p53 backgrounds. At a concentration of 20 µM, JKA97 inhibited the proliferation by about 35%, 35%, and 45%, in MCF7, MDA-MB-468, and MCF7 p53KD cells, respectively.

### JKA97 induces apoptosis of breast cancer cells *in vitro*

In addition to inhibiting proliferation, JKA97 also induced apoptosis in breast cancer cells. As illustrated in Fig. 1D, all three of the examined breast cancer cell lines exhibited a significant increase (P<0.05) in apoptosis following exposure to a 20 µM concentration of the compound. In the MCF7, MDA-MB-468, and MCF7 p53KD cells, a 20 µM JKA97 increased the apoptotic index 3.9-fold, 5-fold, and 4.2-fold, respectively, as compared to that in control cells.

### JKA97 causes G1 phase arrest in breast cancer cells *in vitro*

We also observed that JKA97 inhibited cell cycle progression, leading to arrest in the G1 phase of the cell cycle in a dose-dependent manner in all three cell lines (Fig. 1E). In both MCF7 and MCF7 p53KD cells, even at the 5 µM concentration, JKA97 led to a significant (P<0.05) increase in the number of cells in the G1 phase. At the 20 µM concentration, a majority of the cells were arrested in the G1 phase (P<0.05).

### JKA97 decreases xenograft tumors growth *in vivo*

**Figure 2:**
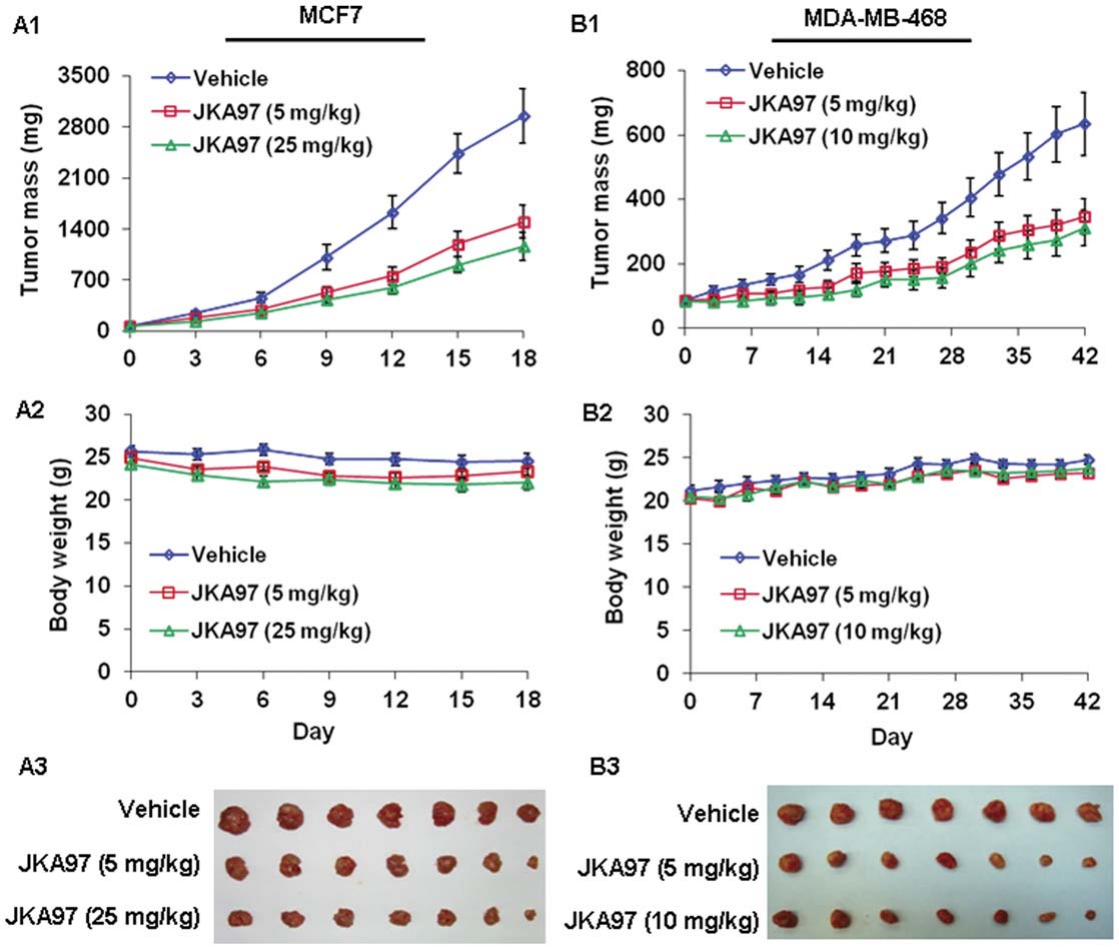
*In vivo* anticancer activity of JKA97 against breast cancer cells. JKA97 was administered by i.p. injection to nude mice bearing MCF7 (A1) or MDA-MB-468 (B1) xenograft tumors. For mice bearing MCF7 xenograft tumor, treatment groups received JKA97 at doses of 5 mg/kg/day or 25 mg/kg/day, 5 days/week, for 6 weeks; for mice bearing MDA-MB-468 xenograft tumor, treatment groups received doses at 5 mg/kg/day or 10 mg/kg/day, 5 days/week, for 18 days. Control groups received vehicle only. Animals were also monitored for changes in body weight as a surrogate marker for toxicity when it was administered to nude mice bearing (A2) MCF7 or (B2) MDA-MB-468 xenograft tumors. At the end of the experiments, xenograft tumors were removed and taken a photograph (A3 and B3).

To determine whether the compound can also exert anti-cancer effects *in vivo*, JKA97 was administered to nude mice bearing MCF7 or MDA-MB-468 xenograft tumors. In the MCF7 xenograft model, the high dose of JKA97 (25 mg/kg) inhibited tumor growth by about 60% (P<0.05) on day 18, with the low dose (5 mg/kg) also leading to significant tumor growth inhibition (50%, p<0.05; Fig. 2A1). The *in vivo* anticancer activity of JKA97 was further investigated in MDA-MB-468 xenograft model. This model appeared to be slightly less sensitive to the drug, with the low dose (5 mg/kg) and high dose (10 mg/kg) decreasing tumor growth by about 45% and 52%, respectively (P<0.05; Fig. 2B1). Additionally, there were no significant differences in body weights between controls and animals treated with JKA97, or any gross organ abnormalities at necropsy in either group (Figs. 2A2 and B2).

### JKA97 up-regulates p21 expression in a p53-independent manner

**Figure 3:**
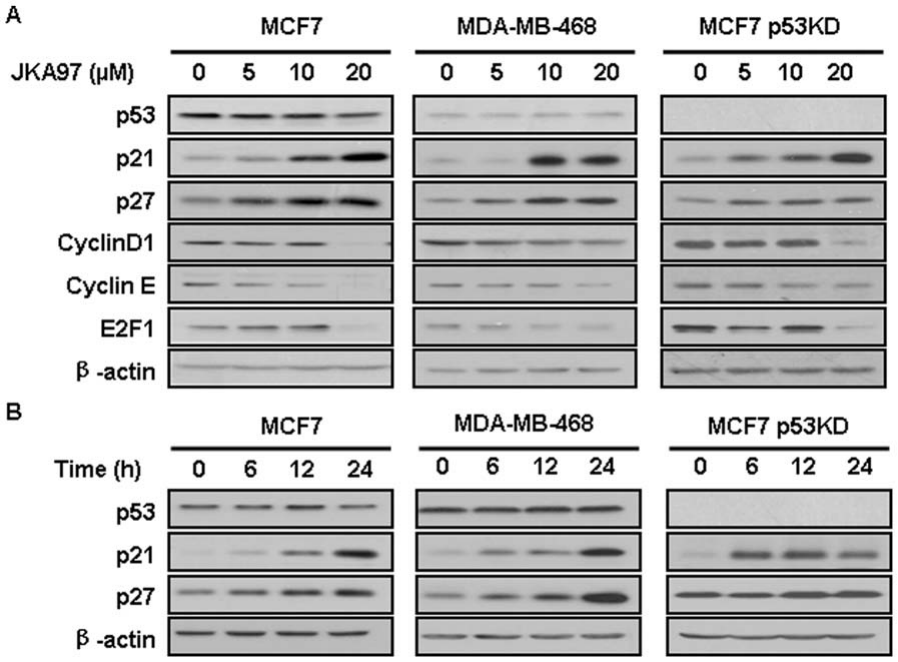
Effects of JKA97 on the expression of cell cycle related proteins. (A) MCF7, MDA-MB-468, and MCF7 p53KD cells were exposed to various concentrations of JKA97 for 24 hrs, and the expression of p53 and p21 proteins was determined by Western blot analysis. (B) Cells were exposed to 10 µM of JKA97 for the indicated time, and the expression of p53 and p21 proteins was determined by Western blot analysis. β-actin was used as an equal-loading control of samples.

We next investigated the mechanism(s) of action of JKA97 by examining its effects on the expression levels of various proteins involved in cell cycle progression. All three cell lines were treated with various concentrations of JKA97 for 24 hrs, Treated cells showed increased p21 expression in a dose-dependent manner. In addition, CyclinD1, CyclinE, and E2F1 were decreased, whereas p27 was increased, most likely due to p21 activation (Fig. 3A). We further treated all three cell lines with 10 µM JKA97 for varying times, as shown in Fig. 3B. The p21 protein levels were increased in a time-dependent manner in all cells tested, regardless of p53 status.

### JKA97 has little effects on the stability of p21 protein

**Figure 4:**
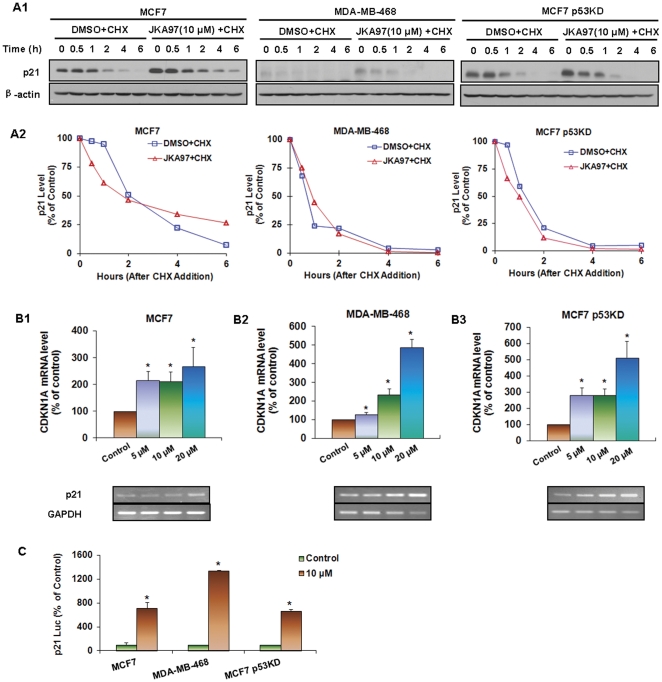
Effects of JKA97 on p21 expression. (A1) MCF7, MDA-MB-468, and MCF7 p53KD cells were exposed to various concentrations of JKA97 or vehicle for 24 hrs, followed by exposure to protein synthesis inhibitor cycloheximide (CHX, 10 µg/mL). p21 protein expression was detected by Western blotting at different times after exposure of CHX. (A2) The graph shows the quantification of the Western blotting data. MCF7 (B1), MDA-MB-468 (B2) and MCF7 p53KD (B3) Cells were exposed to various concentrations of JKA97 or vehicle for 24 hrs, and total RNA were extracted followed by reverse transcription, and detection mRNA level of p21 by Real-time quantification PCR and Quantification RT-PCR, normalized by mRNA level of GAPDH. (C) Cells were transfected with p21 promoter luciferase reporter plasmid and a Renilla luciferase reporter together for 12 hrs, followed by treatment of 10 µM JKA97 or vehicle for an additional 24 hrs. The reporter activity was normalized to the corresponding Renilla luciferase reporter. The luciferase assay was performed in triplicate. Statistical significance was determined compared with control (*P<0.05).

The effects of JKA97 on p21 regulation were also determined at the posttranscriptional level. All three cell lines were exposed to 10 µM of JKA97 or solvent for 24 hrs followed by addition of the protein synthesis inhibitor, cycloheximide (CHX, 10 µM). As shown in Fig. 4A, there was no appreciable difference in p21 protein stability between cells exposed to JKA97 and those exposed only to the solvent.

### JKA97 induces p21 transcription

To determine if p21 upregulation by JKA97 resulted from increased mRNA at the transcriptional level, all three cell lines were treated with various concentrations of JKA97 for 24 hrs, and the p21 mRNA expression levels were determined by real-time PCR. Additional semi-quantification RT-PCR was employed for consistency. As illustrated in Fig. 4B, the expression of p21 mRNA was increased by JKA97 in a dose-dependent manner.

To further demonstrate how JKA97 affects p21 transcription, a human full-length p21 promoter reporter was transfected into all three cell lines, and these cells were then exposed to JKA97 (10 µM). As shown in Fig. 4C, the luciferase activities of the p21 reporter were significant increased by 7.1-fold, 13.4-fold, and 6.6-fold in MCF7, MDA-MB-468, and MCF7 p53KD cells, respectively.

## Discussion

Previous studies indicate that JKA97 induced apoptosis of colon and liver cancer cells by a p53-independent and Bax-dependent pathway [10]. However, other possible mechanisms and the compound's potential for treating other cancer types have not yet been fully elucidated. To the best of our knowledge, the present study is the first to investigate both *in vitro* and *in vivo* anti-tumor effects of the novel harmine benzylidene analog in human breast cancer cells. The study highlights several important points: 1) JKA97 significantly inhibits breast cancer cell growth; 2) JKA97 induces cell apoptosis; 3) inhibition of cell proliferation and cell cycle progression appear to be major mechanism by which JKA97 exerts its anti-cancer effects; 4) JKA97 up-regulates p21 expression at the transcriptional level, rather than post-transcriptional level, independent of p53; and 5) JKA97 decreases the growth of human breast cancer xenograft tumors in mice, in a dose-dependent manner.

Our results indicated that there were remarkable differences in the cell survival after JKA97 treatment, as determined by the MTT assay, with MDA-MB-468 cells being the most sensitive (Fig. 1B). MTT assay represents the total cell survival, which may be affected by various factors, at least including cell proliferation, apoptosis, and cell cycle progression. Therefore, we further determined the effects of JKA97 on cell proliferation, apoptosis, and cell cycle distribution. We used BrdU incorporation assay to determine the cell proliferation index. Although there were dose-dependent effects on cell proliferation in each of the tested cell lines used in this study, there were no remarkable differences between MCF7 (p53 WT) and MDA-MB-468 (p53 Mutant) cells, indicating that the JKA97 effects on cell proliferation are p53-independent. Interestingly, p53 KD MCF-7 cells were more sensitive than other two cell lines (Fig. 1C). However, MDA-MB-468 cells were shown to be more sensitive in apoptosis (Fig. 1D) and cell cycle distribution assay (Fig. 1E). For example, at the highest dose used (20 µM), MCF7 and MCF7 p53 KD cells increased apoptosis similarly, but MDA-MB-468 cells were more sensitive. Taken together, our results indicate that induction of apoptosis and cell cycle arrest are most likely to be related to the differences in cell survival assay among the three cell lines. The detailed mechanisms need to be explored in the future studies.

In the present study, we also demonstrated that JKA97 had significant *in vivo* anti-tumor activity in two breast cancer xenograft models. At the 5 mg/kg dose level, we have observed the similar responses to the therapy between the two models. Since we used different higher doses in MCF7 and MDA-MB-468 models, it is hard to compare the response curves. The dose used in MDA-MB-468 model (10 mg/kg) was less than half of the dose in MCF7 model (25 mg/kg), but generated similar tumor growth inhibition rates. We speculate that MDA-MB-468 cells are more sensitive. Considering that there are other factors affecting the *in vivo* response to JKA97 treatment, we cannot draw firm conclusion regarding the difference between the two models. Further studies are needed to demonstrate such differences and underlying mechanisms.

Obstruction of cell cycle progression in cancer cells is considered as one of the most effective strategies for the control of tumor growth [11]. The shift from a dormant quiescent stage (G0) to an actively growing state is a prerequisite for entry into the cell cycle in most cells and a crucial step for cancer cells. Cell cycle progression is modulated by regulators known as cyclin-dependent kinase (CDK) inhibitors. The first of these proteins to be identified and cloned is p21 [12]–[14]. The p21 protein, a universal cell cycle inhibitor, binds to cyclin-CDK complexes and proliferating cell nuclear antigen, thereby inducing cell arrest at G1 and blocking cell entry into the S phase. Bearing this in mind, we checked the relationships between G1 arrest and cell cycle related regulatory proteins including p53, p21, and p27, after drug treatment. Our results revealed that JKA97-mediated G1 arrest in breast cancer cells was linked with p21, regardless of p53 status (wild-type, mutant, or knockdown). The up-regulation of p21 and p27 proteins enhances the formation of complexes with the G1-S CDKs and cyclins, thereby inhibiting their activities [15]–[17]. This is believed to be the first report to demonstrate the mechanism of p53-independent G1 cell cycle arrest induced by JKA97 in breast cancer cells. Our study further demonstrated that up-regulation of p21 induced by JKA97 was mainly dependent on a transcriptional mechanism rather than a post-translational alteration. Future studies should examine the underlying mechanisms concerning how JKA97 up-regulates p21 transcription and influences the downstream pathways.

Many researchers have suggested that cell cycle arrest and apoptosis may be linked. Molecules acting on cells in G1 phase are normally thought to affect apoptosis; CDK inhibitors have been suggested to be indirectly involved in apoptosis through regulation of CDKs. p21, a major CDK inhibitor, is induced by both p53-dependent and -independent mechanisms following stress [18]. Additionally, it is reported that overexpression of p21 results in an induction of Bax and promotes apoptosis [19]. Considering that *Luo et al.* [10] have demonstrated that JKA97 can induce apoptosis in colon cancer cells in a Bax-dependent but p53-independent manner, further work is required to correlate the relationship between the cell cycle arrest and apoptosis pathways regulated by the compound.

The observations from the present study provided rational evidence for the use of JKA97 as an anti-tumor agent for human breast cancer therapy. Our results, taken in conjunction with earlier results for this compound, indicate that the anti-cancer activity of JKA97 is inextricably linked with modulation of p21. Further studies involving p21 deficient systems would be required to fully gauge the extent of involvement of this important protein. The present study, together with previous reported findings [10], will surely improve our understanding of the mechanisms of action of JKA97, providing a basis for further preclinical and clinical evaluation of the compound as a novel anti-cancer agent.

## Materials and Methods

### Test Compound, Chemicals and Reagents

The test compound JKA97, methoxy-1-styryl-9H-pyrido-[3,4-b]-indole, was synthesized and purified as previously reported [10], and the structure was confirmed by UV, IR, MS, and NMR spectroscopy [10]. The purity of the test compound was determined to be greater than 95% by HPLC and MS analyses. All chemicals and solvents used were of analytical grade or the highest grade available. Cell culture supplies such as culture media, phosphate-buffered saline (PBS), fetal bovine serum (FBS), sodium pyruvate, non-essential amino acids, and penicillin-streptomycin were obtained from Invitrogen (Carlsbad, CA). Antibodies against human p21 (C-19), p27 (C-19), Cyclin D1 (DCS-6), Cyclin E (HE12), and E2F1 (KH95) were from Santa Cruz Biotechnology, Inc. (Santa Cruz, CA). The anti-human p53 antibody (Ab-6) was from EMD Chemicals, Inc. (Gibbstown, NJ). The anti-human β-actin antibody (T5168) was from Sigma-Aldrich (St. Louis, MO).

### Cell lines and Culture

Human breast cancer MCF7 and MDA-MB-468 cells were obtained from the American Type Culture Collection (Rockville, MD). MCF7 and MDA-MB-468 cells were grown in MEM media containing 1 mM non-essential amino acids and Earle's BSS, 1 mM sodium pyruvate and 10 mg/L bovine insulin, and DMEM/F-12 Ham's media (DMEM/F-12 1∶1 mixture). MCF7 p53KD cells were generated by using the method described previously [20], [21] and were grown in the same media as MCF7 cells, but supplemented with 0.5 µg/mL puromycin (Sigma; St. Louis, MO). All cell culture media contained 10% FBS and 1% penicillin/streptomycin, unless otherwise specified.

### Cell Survival Assay

The effects of JKA97 on human breast cancer cell growth were determined by using the MTT assay and expressed as the percentages of control cell survival [22]–[25]. The cells were grown in 96-well plates, seeding at a density of 4–5×10^3^ cells per well, and exposed to various concentrations of the test compound (0 to 50 ìM) for 72 hrs. 10 µL of the 3-(4,5-dimethylthiazol-2-yl)-2,5-diphenyl tetrazolium bromide (MTT) solution (5 mg/mL; Sigma; St. Louis, MO) were then added. The absorbance at 570 nm was recorded by using a SYNERGY Mx microplate reader (BioTek, Winooski, VT). The cell survival rates (%) were calculated by dividing the mean OD of compound-containing wells by that of control wells.

### Cell Proliferation Assay

The effects of JKA97 on cell proliferation were determined by the BrdUrd incorporation assay (Oncogene, La Jolla, CA), following the manufacturer's protocol. Cells were seeded in 96-well plates (8×10^3^ to 1.2×10^4^ cells per well) and incubated with various concentrations of JKA97 (0, 5, 10 and 20 ìM) for 48 hrs. BrdUrd was added to the medium 10 hrs before termination of the experiment. The BrdUrd incorporated into cells was determined by anti-BrdUrd antibody, and the absorbance was measured at dual wavelengths of 450/540 nm with a SYNERGY Mx microplate reader (BioTek, Winooski, VT).

### Detection of Apoptosis

Cells in early and late stages of apoptosis were detected using an Annexin V-FITC apoptosis detection kit from BioVision (Mountain View, CA). In brief, 4–5×10^5^ cells per well were exposed to the test compound (0, 5, 10 and 20 µM) and incubated for 24 hrs prior to analysis. Cells were collected and washed with serum-free media, then re-suspended in 500 µL of Annexin V binding buffer, followed by addition of 5 µL of Annexin V-FITC and 5 µL of propidium iodide (PI). The samples were incubated in the dark for 5 min at room temperature and analyzed with a FACSCaliber flow cytometer (BD Biosciences, San Jose, CA).

### Cell Cycle Measurements

To determine the effects of JKA97 on cell cycle distribution, 4–5×10^5^ cells per well were exposed to the compound (0, 5, 10, and 20 ìM) and incubated for 24 hrs prior to analysis. Cells were harvested and fixed in 75% ethanol at 4°C overnight, followed by incubation with RNAse and staining with propidium iodide (Sigma). The DNA content was determined by flow cytometry as indicated above.

### Mouse Xenograft Model of Human Breast Cancer

The animal use and care protocol was approved by the Institutional Animal Use and Care Committee of the Texas Tech University Health Sciences Center (IACUC # 10032, PHS Assurance # A 3056-01, USDA Registration # 74-R-0050, REF # 039461). Female athymic pathogen-free nude mice (nu/nu, 4–6 weeks) were purchased from Charies River Laboratories International, Inc. (Wilmington, MA). To establish MCF7 human breast cancer xenograft tumor model, each of the female nude mice was first implanted with a 60-day subcutaneous slow release estrogen pellet (SE-121, 1.7 mg 17β-estradiol/pellet) obtained from Innovative Research of America (Sarasota, FL). On the following day, MCF-7 cells harvested from monolayer cultures were washed twice with serum-free medium, re-suspended in the medium, and then injected s.c. (5×10^6^ cells, total volume 0.2 mL) into the left inguinal area of the mice. For the MDA-MB-468 xenograft model, we used the same procedure as above, but without the estrogen pellet. All animals were monitored for activity, physical condition, body weight, and tumor growth. Tumor size was determined every other day by caliper measurement of two perpendicular diameters of the implant. Tumor mass (in g) was calculated by the formula, 1/2*a*×*b* ^2^ where “*a*” is the long diameter and “*b*” is the short diameter (in cm).

### *In vivo* chemotherapy with JKA97

The animals bearing human cancer xenograft tumors were randomly divided into various treatment groups and a control group (7–10 mice/group). The control groups for both models received the vehicle only. For the MCF7 xenograft model, JKA97 was dissolved in the vehicle, PEG400: ethanol: saline (57.1∶14.3∶28.6, v/v/v), and was administered by i.p. injection at doses of 5 and 25 mg/kg/d, 5 days/wk for about 3 weeks. For the MDA-MB-468 xenograft model, JKA97 was administered by i.p. injection at doses of 5 and 10 mg/kg/d, 5 days/wk for 6 weeks. At the end of the experiments, xenograft tumors were removed, weighed, and photographed for records.

### Western Blot Analysis

The protein levels in cell lysates were assessed using Western blotting as described previously [20], [21]. In brief, cells were exposed to various concentrations of JKA97 for 24 hrs and cell lysates were fractionated with identical amounts of protein by SDS-PAGE. The proteins were transferred to Bio-Rad trans-Blot nitrocellulose membranes (Bio-Rad Laboratories, Hercules, CA) that were then incubated in blocking buffer (Tris-buffered saline containing 0.1% Tween 20 and 5% nonfat milk) for 1 hr at room temperature. Then the membranes were incubated with the appropriate primary antibodies overnight at 4°C or 2 hrs at room temperature, with gentle shaking. The membranes were washed with rinsing buffer (Tris-buffered saline containing 0.1% Tween 20) thrice for 15 min and then incubated with the goat anti-mouse/-rabbit IgG-horseradish peroxidase-conjugated antibody (Bio-Rad) for 1 hr at room temperature. After washing thrice, the proteins of interest were detected by using enhanced chemiluminescence reagents from PerkinElmer LAS, Inc (Boston, MA).

### RNA extraction, and semi-quantitative and real-time quantitative RT-PCR

Total RNA was extracted by using the Trizol reagent from Invitrogen (Carlsbad, CA). First-strand cDNA was synthesized with 1 µg of total RNA extract using the iScript cDNA synthesis kit (Bio-Rad, Hercules, CA). The primer sequences used for amplification of genes were as follows: p21 forward, 5′-AGC AGC GGA ACA AGG AGT-3′, reverse, 5′-TGG AGA AAC GGG AAC CAG-3′; GAPDH forward, 5′-GGA GTC CAC TGG CGT CTT CAC-3′, reverse, 5′-GAG GCA TTG CTG ATG ATC TTG AGG-3′. Semi-quantitative RT-PCR was performed with mixture of the cDNA product, primers, dNTP and Taq DNA polymerase (Invitrogen) using cycles of 95°C for 30 s, 57°C for 30 s, and 72°C for 30 s, followed by a final extension at 72°C for 5 min. Real-time PCR was performed for 40 cycles consisting of 95°C for 20 s, 57°C for 20 s and 72°C for 20 s using an iQ5 machine (Bio-Rad, USA). All samples were analyzed by the comparative threshold cycle (C_T_) method and normalized to the GAPDH mRNA control.

### Luciferase Assay

Cells were cotransfected with full-length human p21 promoter vectors with *Renilla* luciferase reporter (as internal control; Promega, Madison, WI) for 24 hrs, followed by incubation with JKA97 for 24 hrs. The luciferase activity of the p21 promoter reporter was determined with the Dual-Luciferase Reporter Assay System (Promega, Madison, WI) according to the manufacturer's instructions. The p21 reporter activity was normalized to the corresponding *Renilla* luciferase reporter activity.

### Statistical Analysis

Data of different treatment groups are presented as means ± standard errors. One-way ANOVA followed by S-N-K post hoc test was used to determine the significance of the differences among treatment groups and controls. Results were considered statistically significant when P<0.05.
